# Secretome Proteomic Approaches for Biomarker Discovery: An Update on Colorectal Cancer

**DOI:** 10.3390/medicina56090443

**Published:** 2020-08-31

**Authors:** Armando Cevenini, Stefania Orrù, Esther Imperlini

**Affiliations:** 1 Dipartimento di Medicina Molecolare e Biotecnologie Mediche, Università degli Studi di Napoli “Federico II”, 80131 Napoli, Italy; armando.cevenini@unina.it; 2 CEINGE-Biotecnologie Avanzate S.c.a r.l., 80145 Napoli, Italy; stefania.orru@uniparthenope.it; 3 Dipartimento di Scienze Motorie e del Benessere, Università “Parthenope”, 80133 Napoli, Italy; 4 IRCCS SDN, 80143 Napoli, Italy

**Keywords:** secretome, colorectal cancer, cancer cell lines, biomarker, proteomics, mass spectrometry

## Abstract

Searching for new cancer-related biomarkers is a key priority for the early detection of solid tumors, such as colorectal cancer (CRC), in clinically relevant biological fluids. The cell line and/or tumor tissue secretome represents a valuable resource for discovering novel protein markers secreted by cancer cells. The advantage of a secretome analysis is the reduction of the large dynamic range characterizing human plasma/serum, and the simultaneous enrichment of low abundance cancer-secreted proteins, thereby overcoming the technical limitations underlying the direct search in blood samples. In this review, we provided a comprehensive overview of recent studies on the CRC secretome for biomarker discovery, focusing both on methodological and technical aspects of secretome proteomic approaches and on biomarker-independent validation in CRC patient samples (blood and tissues). Secretome proteomics are mainly based on LC-MS/MS analyses for which secretome samples are either in-gel or in-solution trypsin-digested. Adequate numbers of biological and technical replicates are required to ensure high reproducibility and robustness of the secretome studies. Moreover, another major challenge is the accuracy of proteomic quantitative analysis performed by label-free or labeling methods. The analysis of differentially expressed proteins in the CRC secretome by using bioinformatic tools allowed the identification of potential biomarkers for early CRC detection. In this scenario, this review may help to follow-up the recent secretome studies in order to select promising circulating biomarkers to be validated in larger screenings, thereby contributing toward a complete translation in clinical practice.

## 1. Introduction

Early detection through specific molecular markers is still a key step affecting the clinical management of cancer. In fact, despite the remarkable knowledge acquired on cancer biology and on pharmaceutical therapies, if the tumor is detected in its final stages, the outcome is still unfavorable. In such a framework, colorectal cancer (CRC) is found. CRC is the third most commonly diagnosed cancer in males, the second in females, whose incidence varies greatly worldwide: the highest rates are in high-income countries (North America, Europe) and the lowest in Africa and South-Central Asia [1,2]. This variability is due not only to genetically determined susceptibility, but an unhealthy lifestyle (physical inactivity, unbalanced diet, smoking, obesity) and adverse environmental exposures (such as food colorants and preservatives, antibiotics in agriculture and medicine) may also play a crucial role in CRC development [2,3,4]. 

Over the last 40 years CRC incidence has decreased among adults aged over 50, following the changing patterns in risk factors and the wide application of stool-based tests and colonoscopy screening [5]. Meanwhile, incidence rates have raised among those younger than 50 (2%/year since 1994) [2,4,6]: some of these early-onset cases could be due partly to hereditary CRC syndromes (about 30%) or family history of CRC (about 20%), but for many of them (about 50%) the causes are still unknown [3,4,6,7]. The CRC therapeutic protocols encompass a cocktail of oxaliplatin (oxa), 5-fluorouracil (5-FU) and folinic acid (FOLFOX) or capecitabine (XELOX), and their duration strongly depends on CRC stage and clinical parameters of the patients [8].

Over time, many attempts have been performed to find cancer-related molecular biomarkers, capable of early detection of CRC or other solid tumors, in clinically relevant biological fluids (such as blood or urine) [9]. The main drawbacks are related to the heterogeneity of the biological matrix and its very high dynamic range of concentrations among species (up to 12 orders of magnitude in plasma/serum): such a complexity represents a challenging task (depletion of highly concentrated species, sample pooling) when trying to reliably detect low abundance cancer-related biomarkers [10,11,12,13,14,15,16,17]. Lately, these experimental limits, along with the difficulties of translating the results into clinical applications, have discouraged researchers from following that path [11].

When looking for such biomolecular markers, it was evident that the search should not be limited to tumor intracellular species, but be extended to the tumor microenvironment enriched by secreted proteins from the cancer and cancer-related cells, under specific conditions and time [18]. As a matter of fact, the local tumor milieu is a very complex system characterized by different types of cells, apart from tumor cells: mesenchymal stem cells, adipocytes, cancer-associated fibroblasts (CAFs), endothelial cells, tumor-infiltrated lymphocytes, inflammatory cells and macrophages [19]; each of them is engaged in a reciprocal interaction, by secreting specific factors while being influenced by others [20,21]. This multidirectional interplay represents a well-orchestrated network of autocrine and paracrine signals aimed at tumor growth and at remodeling of the tumor microenvironment [22,23,24,25,26]. The full set of components secreted by such a microenvironment, including extracellular matrix proteins, enzymes, growth factors, inflammatory cytokines and exosomes and microvesicles, constitutes the cancer cell secretome, an invaluable source of circulating biomarkers [10,27]. In addition, as cancer results from the multiple accumulating abnormalities in the genome (such as mutations, deletions, insertions and chromosomal translocations), this specific milieu is also characterized by degraded protein products, coming from cancer-related mutated species, which could be extremely powerful in their diagnostic potential [28]. Accordingly, in the last decade, secretome analysis has gained interest as the tumor interstitial fluids (TIFs) are semi-complex mixtures, particularly enriched in cancer-related factors but lacking high-abundance interfering species, that are effortlessly transferred to any experimental protocol and easily mimicked by conditioned media (CM) of cancer cell cultures [9,18,29].

In this review, we attempt to shed light on current trends in proteomic applications to secretome for biomarker discovery with a focus on CRC. In particular, the key steps of the biomarker discovery protocols in CRC secretome are discussed, including the sample types, the sample preparation, the mass-spectrometry (MS)-based methods (label-free quantitative proteomics and gel-based, affinity-based and shotgun approaches) and bioinformatic analysis. Finally, the emerging data from the most recent publications are overviewed to point out the potential of secretome analysis for the identification of new circulating biomarker candidates.

## 2. Secretome Samples

### 2.1. Conditioned Media from CRC Cell Lines

The conditioned media (CM) from the different available CRC cell lines is the most analyzed secretome sample. In addition to being xenograft tumor models for CRC, human colon adenocarcinoma cell lines, such as CaCo-2, LIM1215, HCT-116 and HT-29, are used as in vitro CRC model systems to analyze intracellular and secreted proteins from intestinal epithelial cells [30,31,32,33,34,35,36,37,38,39,40,41,42].

CaCo-2 spontaneously differentiates into mature enterocytes, and at confluence, forms a polarized monolayer with the specific functional features of differentiated absorptive epithelial cells [42]. LIM1215 represents a moderately differentiated CRC cell line but a metastatic one unlike CaCo-2 [43]. On the contrary, HCT-116 differentiation is to be modulated; interestingly, undifferentiated HCT-116 cells can be compared with their metastatic derivative counterpart to study the tumor phenotype [38,40]. Similarly to HCT-116, HT-29 cells under standard culture conditions are also undifferentiated, but upon differentiation induction they acquire a polarized morphology [44]. 

The majority of secretome studies are focused on one or two CRC cell lines; however, there are few integrative studies on the secretome analysis of multiple CRC cell lines recapitulating the differences in differentiation status and thus reflecting the biological variability within CRC [45,46].

CM collection from selected CRC cell lines, upon 70–80% confluency, is performed from serum-free cultures. After 24–48 h, depending on the experimental design, the CM is centrifuged, filtered and further processed for secretome analysis. A critical step is to obtain CM samples without intracellular protein contaminants, which are released in the media under cellular stress conditions. In this regard, following the CM collection, it is recommended to check the cell viability and/or the lactate dehydrogenase (LDH) levels: in fact, counting viable cells via conventional assays and measuring low LDH levels (if not its absence) indicate negligible cell death and insignificant cytoplasmic contamination in secretome preparations [44].

Alternatively, the hollow fiber culture (HFC) system could be used to reduce cell death and detect low abundance proteins in the large volumes of collected CM [47]. This system, composed of small fibers sealed in a cartridge shell, provides an in vivo-like 3D environment able to hold many cells in a small volume, thereby concentrating the secreted proteins. The HCF system has been successfully used for secretome analyses of CRC [38] and other solid cancers [48]. 

### 2.2. CRC Tissue Secretome

In comparison with CRC CM analyses, to the best of our knowledge, there are few reports on CRC tissue secretomes [31,49,50,51]. As stated above, tumor tissue secretome and particularly TIFs are promising sources of CRC biomarkers, represented by proteins secreted in vivo in the tumor microenvironment. The advantage of TIFs, in fact, is the reduction of the large dynamic range characterizing human plasma/serum, and the simultaneous enrichment of tumor-secreted proteins. After collection and washing of CRC tissue specimen samples together with their normal counterparts (adjacent normal colon mucosa), they are sliced into 1–3 mm^3^ pieces and incubated in physiological medium, such as PBS, at 37 °C for 1 h. After high-speed centrifugation, the supernatant represents the tissue secretome to be further analyzed [49]. 

Otherwise, freshly isolated CRC tissue specimens can be cultured ex vivo as explants and their relative secretome preparations are mainly composed of TIFs proteins secreted by tumor cells [52]. In this context, samples widely employed in discovering CRC biomarkers, are the CM of cancer-associated fibroblasts (CAFs) isolated from fresh surgical CRC specimens and adjacent normal tissues [39,53,54]. Such a sample is suitable for analyzing the autocrine factors from CRC cells and paracrine factors from surrounding stroma cells, mostly formed by fibroblasts. Interestingly, the interaction of stroma cells with tumor cells, underlying the carcinogenesis and metastatic process, is mediated by soluble proteins released in the CM of cell co-cultures. 

In addition to CM from co-culturing CRC cells with stroma cells, Bozzi and colleagues performed a secretome analysis of CRC organoid cultures of peritoneal metastatic lesions from one CRC patient. In serum-free cell culture conditions, CRC organoids are suitable materials for the identification of secreted biomarkers involved in cancer stem cell (CSC) self-renewal and cancer cell proliferation [55]. 

To study the mechanisms underlying tumorigenicity and/or drug resistance in CSCs, the proteins secreted from the CM of stem cells were identified after their isolation from human CRC tumors; their expansion in derived cell cultures and in vitro differentiation were also explored [56]. 

## 3. Secretome Proteomic Approaches

Current secretome studies are mainly performed by unbiased label-free qualitative and quantitative proteomics analyses based on LC-MS/MS methods for which secretome samples are either in-gel or in-solution trypsin-digested (Table 1). All these procedures surely enable one to analyze multiple experimental conditions within the same study design to ensure high reproducibility; however, it is instrumental to consider an adequate number of biological and technical replicates for condition. 

### 3.1. Label-Free LC-MS/MS Proteomics

A classical qualitative bottom-up proteomic gel-based workflow was used for identification of secreted proteins in the serum-free CM from different CRC cell lines and CAFs and in CRC tissues and their normal counterparts (Table 1). As for CM samples, after their collection, proteins are analyzed following a typical gel-based MS protocol (SDS-PAGE/in-gel tryptic digestion/nanoLC-MS/MS). As an example, by using this approach, Chen and colleagues analyzed two pairs of CAF/normal counterparts from two CRC patients, running each sample twice [54]. The combination of a limited number of biological/technical replicates coupled to a traditional gel-based proteomic approach, allowed to unambiguously identify only 230 proteins (at a false discovery rate of 1.3%). Moreover, the comparison of these data with a previously published dataset [53] produced poor overlapping, probably due to the biological variability among fibroblasts isolated from different CRC patients and also different experimental/technical conditions. On the contrary, a similar gel-based MS protocol performed on the secretome of CRC tissues and their normal counterparts led to the identification of 2703 unique proteins; the improvement in terms of uniquely identified species was related both to the sample type (tissue) and the availability of a suitable number of biological replicates (*n* = 4). Moreover, among the four biological replicates, the best overlapping in terms of commonly identified protein species was observed in the four CRC tissues rather than in the four patient-matched cancer-normal colon tissues, highlighting the importance of properly enrolling patients with matching clinicopathological features. Importantly, these identified proteins were then compared with those found in the secretomes of five typical CRC cell lines analyzed by the same authors and in the same experimental conditions of tissue samples [49]. This enlightened step clarified that secretory proteins identified in CRC tissue secretomes arose from neoplastic epithelial cells rather than the surrounding tumor environments [49].

In addition to the prefractionation by gel electrophoresis of CM samples, differential centrifugation ultrafiltration can be used to separate secretome components into molecular weight (MW)-based fractions (Table 1) [35,36]. Medium and high-MW (3–30 KDa and >30 KDa) secreted protein fractions were analyzed by the classical bottom-up proteomics gel-based workflows as described above. Interestingly, low-MW (1–3 KDa) soluble peptide fractions (peptidome) were subjected to a top-down proteomic approach [57]. All fractions from the bottom-up and top-down approaches were analyzed by nanoLC-MS/MS, and as expected, each approach showed distinct features in terms of identified protein/peptide species [36].

### 3.2. Label-Free LC-MS/MS Shotgun Quantitative Proteomics

In addition to the reproducibility and robustness of secretome studies, label-free proteomic quantitative analysis guarantees elevated accuracy through spectral counting (SpC) and/or precursor ion intensity (total ion counts, TIC) methods (Table 1). To quantify all species present in secretome samples it is appropriate to perform both SpC and TIC methods as reported by Greening and colleagues; these approaches, in fact, complement each other because SpC and TIC procedures provide accurate estimations for high- and low abundance proteins, respectively [35].

As for reproducibility and accuracy of secretome analysis, the label-free LC-MS/MS shotgun quantitative proteomics can be considered a high-performing protocol; in particular, the CM from CRC cell lines is subjected to in-solution tryptic digestion and subsequently analyzed, at least in triplicate, using, in general, a bidimensional chromatography (cation exchange, SCX and reversed phase, RP) coupled to MS/MS.

As reported by Karagiannis and colleagues, this system led to 2979 unique proteins identified with a minimum of two peptides and FDR < 0.1%, and with a high reproducibility (>60%) among the triplicates [46]. Moreover, an innovative aspect of the quantitative approach performed by Karagiannis et al. regarded the introduction of an internal control, consisting of a panel of four known extracellular proteases. Their protein abundance was estimated both by ELISA and SpC: the statistically significant correlation among the two methods provided a validation of the label-free quantitative analysis [46]. 

In this context, label-free quantitative sequential windowed acquisition of all theoretical fragment ion (SWATH)-MS technology has been recently reported as a highly reproducible and sensitive method for CRC biomarker discovery [38,48]. SWATH-MS is a data independent acquisition (DIA) method that combines shotgun and selected reaction monitoring (SRM) analyses; it consists of the collection of high-resolution fragment ion maps of all detectable peptides present in the analytical sample. These peptides within specified precursor mass and retention time ranges are systematically unbiased fragmented [58]. SWATH-MS data analysis requires the in-advance creation of a spectral library in data dependent acquisition (DDA) of all the detectable peptides in the samples [59]. This technology was successfully applied to compare glycosecretomes of HCT-116 cell line with its metastatic derivative E1 [38]. Multilectin affinity chromatography (MLAC), in fact, is a suitable method to enrich glycoproteins into secretome samples, thereby enhancing the detection of low abundance CM-secreted proteins [38]. Recently, the study of glycosecretome has been gaining popularity because of the potential involvement of glycosylation modifications along the secretory pathway in intestinal epithelial cell differentiation. In this context, Link-Lenczowski and colleagues qualitatively and quantitatively analyzed chemical labeled-N-oligosaccharides from undifferentiated and differentiated CaCo-2 cells by using hydrophilic interaction liquid chromatography (HILIC)-high performance liquid chromatography (HPLC) and matrix-assisted laser desorption/ionization-time of flight-MS (MALDI-ToF-MS) approaches, respectively [42].

### 3.3. Quantitative Proteomics by Labeling Methods

In addition to label-free approaches, metabolic (stable isotope labeling by amino acids, SILAC) [60,61] and chemical labeling (dimethyl labeling, iTRAQ) [62,63] were also applied for quantitative CRC secretome analysis (Table 2). 

In the SILAC-based quantitative proteomics strategy, CRC cell lines are grown on heavy- (^13^C6-Lys/^13^C6-Arg) and light (^12^C6-Lys/^12^C6-Arg)-labeled medium under standard conditions until the confluency is reached. If two types or two different conditions of CRC cell lines are quantitatively compared, it is important to swap the labeled medium to avoid any bias in the labeling. As described above for CM collection, cells are washed and then cultured in the corresponding light- and heavy-labeled serum-free medium; CM are generally harvested after 48 h, concentrated and quantified. Protein extracts from heavy- and light-labeled cells are mixed in molar ratio of 1:1 and subjected to gel-based or shotgun proteomic analyses [32,33,40].

As for chemical labeling, the typical workflow includes the following steps similar to the metabolic one: (a) trypsin digestion of CM; (b) peptide labeling with heavy or light isotopes of a chemical compound; (c) mixing (1:1) of light/heavy peptide samples; and (d) proteomic analysis.

For all labeling methods, specific software, such as Proteome Discoverer and Maxquant, is used to calculate peptide ratios by comparing the intensities of the light- and heavy-labeled precursors at high resolution. In addition, light- and heavy-peptides unpaired in the mass spectra should be checked in the quantitative analysis [40].

In comparison to other classical quantitative proteomic approaches, SILAC-based strategy guarantees higher robustness and accuracy, but also flexibility with current experimental designs. In this case, however, an adequate number of experimental replicates improves the accuracy of quantitative data. The need for high reproducibility is a concept more and more crucial for secretome datasets compared to intracellular ones due to the lower abundance and complexity of secreted proteins to be quantified.

### 3.4. Affinity-Based Proteomics

Given that the cancer secretome is a reservoir of tumor-associated antigens (TAAs), affinity strategy coupled to MS-based proteomics could be a promising approach for capturing immunogenic biomarkers directly from sera of CRC patients, but its intrinsic limit is related to the specificity of the antibody development. In fact, there has only been one recent report on the immune-affinity-MS approach for biomarker discovery in the CRC field [44]. Bukkhari and colleagues generated a polyclonal antibody repertoire against the secretome of HT-29 cell line. This affinity reagent (as immobilized anti-Sc antibody) was used for immunoprecipitate TAAs, whose identification in CRC patient sera compared to the normal sera was performed by a classical gel-based proteomic approach. 

### 3.5. Bioinformatic Analysis of Secretome Datasets

The most widely used bioinformatic tools for qualitative secretome analysis of MS/MS datasets are Mascot (http://www.matrixscience.com) [64], SEQUEST [65] and X!Tandem (https://www.thegpm.org/TANDEM/) [66]. 

In addition, to identify successfully degraded protein products, coming from cancer-related mutated species, and hence to provide a functional connection between genomic and proteomic data in cancer, specific bioinformatics resources, containing comprehensive variant proteins, need to be queried. For example, the Cancer Proteome Variation Database (CanProVar, http://canprovar2.zhang-lab.org) [67,68] contains more than 150,000 cancer-related variations and almost 10,000 cancer-related differentially expressed proteins associated with 26 cancers. Another freely-available tool is the Single Amino Acid Polymorphism Database (dbSAP, http://119.3.41.228:8080/dbSAP/index.html) [69] that integrates data from eight distinct databases and contains more than 16,000 unique variant peptides supported by more than 400,000 MS spectra. Finally, it is possible to query the database of differentially expressed proteins in human cancer (dbDEP 3.0, https://www.scbit.org/dbdepc3/) [70] based on MS data that contains information on amino acid variations, post-translational modifications and drugs.

Another major step during the experimental design of secretome studies is the biological and functional characterization of identified secreted proteins. Subsequent to MS analysis, secretome protein datasets are analyzed by using different bioinformatic tools to gain insights into their structural and functional annotations.

Firstly, secretory features of identified proteins are investigated by using SignalP (http://www.cbs.dtu.dk/services/SignalP/) [71], SecretomeP (http://www.cbs.dtu.dk/services/SecretomeP) [72] and TMHMM (http://www.cbs.dtu.dk/services/TMHMM) [73] tools; they are able to predict whether a protein is classically (SignalP) or non-classically (SecretomeP) secreted and presence of transmembrane helices (TMHMM). For each tool, specific cutoffs have to be considered in order to classify a protein as secreted species; otherwise, for those of exosomial origin, their putative protein annotations can be determined by querying databases such as ExoCarta (http://www.exocarta.org) [74] and Vesiclipedia DB (http://www.microvesicles.org/) [75]. 

In addition to determination of secretory pathways among the experimental dataset, clustering of secreted species into functional annotation terms is usually conducted in secretome studies. To accomplish this, over-represented proteins are identified in datasets through enrichment analysis in Gene Ontology (GO) annotations for molecular function and biological process. GO information is retrieved from different databases such as IPA database (Ingenuity^®^ Systems, www.ingenuity.com) [76] and UniProt DB (www.uniprot.org) [77] and from GO resources (http://geneontology.org) [78,79], and is then analyzed by using several bioinformatic tools such as DAVID (http://david.abcc.ncifcrf.gov/) [80,81], GeneMania (http://www.genemania.org/) [82], PANTHER (http://www.pantherdb.org) [83,84] and BiNGO (http://www.psb.ugent.be/cbd/papers/BiNGO/Home.html) [85]. Predicted protein–protein interactions are investigated using STRING (www.string-db.org) [86], whereas Cytoscape (www.cytoscape.org) [87] tool is used for network analysis. 

The identification of over-/under-represented proteins in CRC secretome versus control by using bioinformatic tools, together with the overlap analysis with previously published similar datasets, allowed the selection of potential biomarkers for early CRC detection.

## 4. Biomarker Discovery for CRC Early Diagnosis by Secretome Proteomics

As carcinogenesis is a long process, early tumor detection requires highly sensitive and specific screening tests whose efficacy relies on the proper biomarkers, mainly in the case of asymptomatic tumors such as CRC [88]. In this context, carcinoembryonic antigen (CEA) and carbohydrate antigen 19-9 (CA 19-9) are the most adopted blood-based biomarkers in current CRC clinical practice, although they are not suitable for early diagnosis [88,89]. Hence, the pre-selection of potential circulating biomarkers is still an ongoing task and secretome proteomic studies fulfill this requirement, thereby overcoming the technical limitations underlying the direct search in clinically relevant biofluids [9].

As stated above, secreted protein biomarkers from CM of CRC cell lines could be detectable candidates in blood. The results from the most recent secretome proteomic studies, including protein biomarkers already related to CRC detection, are shown in Table 1 and Table 2 and discussed below.

We previously reported the secretome signatures of two CRC cell lines (CaCo-2 and HCT-GEO), and among the identified proteins, more than a half were classified as secretory by in silico analysis [34]. Based on the results, we suggested that HCT-GEO CM is enriched by pro-invasive factors, whereas CaCo-2 CM was characterized by adhesion proteins [34]. 

Likewise, Greening and colleagues compared the protein profile of LIM1215 CM with or without sulindac treatment (a chemopreventive nonsteroidal antinflammatory drug) [35]; downregulated proteins in the dataset represented sulindac-sensitive species that were enriched in the CRC secretome. Among the downregulated extracellular matrix (ECM)-remodeling-associated proteins, the authors identified collagens, the basement membrane laminin receptors and several proteoglycans [35]. In another report, the same authors analyzed LIM1215 and LIM1863 secretopeptidomes, and identified, after in silico analyses, secreted proteins implicated in tumor progression and angiogenesis; cell–cell recognition and signaling; and tumor invasiveness and metastasis [36]. 

A wider secretome proteomic analysis was performed by Fanayan and colleagues, who considered 21 CRC cell lines (LIM1215, LIM1863, LIM1899, LIM2099, LIM2405, LIM2463, LIM2537, LIM2550, LIM2551, HCT-15, HCT-116, HT-29, CaCo-2, HCA-7, LOVO, LS174T, SW480, SW620, SW1222, SW1463 and T84). These authors identified about 2500 non-redundant proteins, and using bioinformatic analysis they selected 15 putative CRC-related protein biomarkers, including carcinoembryonic antigen-related cell adhesion molecule 6 (CEACAM6/CD66c) [45]. Interestingly, CEACAM6/CD66c was tested as a CRC stemness marker in CRC patients [90], and recently, the *CEACAM6* transcript was validated as a blood marker for CRC screening, along with the transcripts of *LGALS4*, *TSPAN8* and *COL1A2*, also to discriminate false-positive fecal immunochemical test (FIT) subjects [91].

Karagiannis and colleagues analyzed and compared the secretomes of 12 different CRC cell lines (SW1116, SW480, LS174T, LS180, WiDR, SW620, RKO, LoVo, HCT-116, DLD1, Colo320HSR and Colo205), thereby identifying secreted proteins involved in cell adhesion machinery during cancer progression, especially during phenotypic reprogramming such as epithelial-to-mesenchymal transition (EMT) [46]. A profile of enriched biomarkers of CRC progression was obtained by using a multi-step bioinformatic analysis [46]. Among the selected biomarkers, the levels of olfactomedin-4 (OLFM4) showed a significant correlation with CRC when measured in the sera of CRC patients versus controls. Interestingly, among the proposed biomarkers, there was also carbonic anhydrase IX (CA9), a well-known high expressed isoenzyme in solid tumors [92,93].

By comparing the secretomes of HCT-8 and HCT-116 cell lines with a previous characterized CRC tissue proteome [94,95,96], Shin and colleagues selected 11 secreted candidate biomarkers (Table 1). Interestingly, melanotransferrin (TRFM) plasma levels were found significantly higher in CRC patients compared with healthy controls by using Western blot [37]. This striking result was then confirmed by ELISA in a wider CRC patient group, highlighting for TRFM a very high positive predictive value and very high specificity, and suggesting its suitability for early-stage diagnosis in CRC [37].

Given the positive correlation between immunoglobulin-like cell adhesion receptor L1 (L1CAM) expression and CRC tumorigenesis [97], Basu and colleagues recently found, in L1CAM-overexpressing CRC cells, more than 10-fold increased levels of nine secreted proteins [41]. Among these, they confirmed that increased levels of cathepsin D (CTSD) enhance the motility, tumorigenesis and liver metastasis of CRC cells by activation of Wnt/β-catenin signaling. In particular, high levels of CTSD were found in the invasive front of CRC tumor tissues, thereby suggesting CTSD as a promising biomarker for CRC progression [41]. 

Barderas and colleagues compared the secretome of the KM12C cell line with that of its metastatic counterpart KM12SM, and after bioinformatic analysis they selected 80 potentially secreted and differentially expressed proteins linked to metastatic processes in CRC. Among them, eight proteins were validated in metastatic cells by western blot [32]. The authors also demonstrated by functional experiments that some of them were implicated in cellular adhesion, migration/invasion and metastatic spreading towards the liver in vivo. Interestingly, neuroserpin (SERPINI1), growth/differentiation factor 15 (GDF15) and calcium-binding protein A8/A9 (S100A8/A9) showed potential as biomarkers for CRC diagnosis. In fact, these candidates were validated by ELISA in serum, and they allowed to discriminate CRC patients from healthy controls with high sensitivity and specificity [32].

To investigate the influence of an in vitro-mimicked tumor microenvironment on the protein expression profile, Zeng and colleagues compared the secretomes of HT-29 cell line and of a normal human colon mucosal epithelial cell line (NCM460) alone and in co-cultures [33]. They selected insulin-like growth factor-binding protein 6 (IGFBP6), vimentin (VIM) and acrogranin (GRN) as proteins potentially implicated in the CRC progression and validated them by Western blot, ELISA and immunofluorescence (Table 2). Indeed, the authors pointed out that the changes in the levels of these three proteins correlated with different NCM460/HT29 co-culture ratios resembling different stages of CRC [33]. Interestingly, VIM, besides being a key regulator of cell adhesion and cell–cell interactions [98], has a well-known role in the EMT process in many types of cancer [99], while IGFBP6 is particularly implicated in the IGF1-mediated EMT [33,99,100]. Additionally, Bukhari and colleagues proposed VIM as CRC circulating biomarker by using an affinity proteomic approach as described above. This approach allowed the identification of potential plasmatic cancer biomarkers that might be responsible for CRC development and progression; among these proteins, very high levels of VIM were found in CRC patient sera compared to in healthy controls, and there was a significant correlation of these levels with CRC, thereby supporting the predictive value of VIM for monitoring subjects at increased risk of CRC [44]. 

Until now, there have been few reports on CRC glycosecretome that represent suitable sources of biomarkers using glycoprotein enrichment to detect low abundance secreted proteins in the CM of CRC cell lines. In this context, Bernhard and colleagues analyzed the glycosecretome in LIM1215 CM and in the TIF of tumors derived by the same cell line xenografted in immunodeficient mice. These authors found three secreted species shared between the two type of samples (Table 1). Among these proteins, cadherin-17 (CDH17) was also validated by Western blot in cell lysates, CM, TIFs and in the plasma of xenografted mice, thereby suggesting its potential as a CRC biomarker [31]. A different set of glycoproteins was highlighted by Lin and colleagues when they compared the glysecretome of HCT-116 cell line with its metastatic derivative. A special focus was set on laminin β-1 (LAMB1), over-secreted in the metastatic cell line, which was significantly higher in the sera of CRC patients compared with healthy controls. Moreover, ROC analyses showed that LAMB1 discriminated CRC patients from controls better than CEA [38]. In a recent glycosecretome study, Link-Lenczowski and colleagues highlighted the N-glycosylation changes between two differentiation stages of CaCo-2 cell line. In particular, they found an enhanced fucosylation in differentiated cells and suggested that H4N5F1 glycans might be a biomarker of intestinal epithelial cell differentiation [42].

Some authors focus their attention on CSCs, and hence, on their secretome to fish for the proper CRC biomarkers. At this regard, when Emmink and colleagues compared the secretomes of CSCs and of isogenic differentiated tumor cells (DTCs), isolated from three different metastatic CRCs, most CSC secreted proteins were involved in cell survival, antioxidant activities and proteome integrity maintenance processes [56]. Among them, aldehyde dehydrogenase 1 (ALDH1A1) and bleomycin hydrolase (BLMH) conferred to CSCs resistance against maphosphamide and bleomycin, respectively [56].

In the search for CRC stemness markers, De Boeck and colleagues compared the secretomes of CAFs from two CRC patients with mesenchimal stem cells (MSCs) from bone marrow of healthy individuals [53]. The in silico analysis of the unique species, exclusively identified in CRC CAFs, suggested their involvement in the regulation of cellular movements and cell-to-cell signaling and interactions, and in the inflammatory responses and cancer progression (Table 1). The authors evaluated also the MSC secretome after TGF-β1-induced differentiation, a treatment triggering the conversion of MSCs into CAF precursors: accordingly, they found 16 secreted species shared with CAF secretome including chemokines and growth factors [53].

Qiao and colleagues analyzed the secretomes of five CRC CAFs and of three CRC cell lines and focused on the proteins which were exclusively secreted by CAFs. Among these proteins, COL6A3 was also analyzed by immunohistochemistry (IHC) analysis on a tissue microarray (TMA) containing 90 pairs of CRC and normal counterpart tissues [39]. This analysis showed that COL6A3 protein levels were significantly higher in the stromal cells of CRC tissues than in the normal counterparts (Table 2). Moreover, COL6A3 expression in cancer stroma was correlated to a poor outcome with a significant prognostic value. An ELISA assay also permitted a comparison of COL6A3 plasma concentrations in CRC patients and healthy individuals. This analysis showed COL6A3 upregulation in the CRC patients and demonstrated a very high prediction value, sensitivity and specificity of COL6A3 as plasmatic biomarker of CRC [39].

As for the tissue samples, de Wit and colleagues analyzed the secretomes of CRC tissues paired with their normal counterparts from four CRC patients and the secretomes of five CRC cell lines (Table 1). By performing a multistep bioinformatic analysis where previously reported datasets were taken into consideration [101,102], 21 potential biomarkers were selected for early detection of CRC [49]. This selection included many nuclear proteins involved in DNA replication, cell division and other CRC-related processes. Among them, CRC overexpression of minichromosome maintenance complex component 5 (MCM5) was validated by immunohistochemical staining in a TMA containing 82 colon adenomas and 82 CRCs [49].

Wang and colleagues, instead, generated an inflammation-related CRC mouse model and analyzed the protein profile of TIFs from mouse colon tissues during four stages of CRC development. They focused on 11 tumor growth-related proteins analyzed by multiple reaction monitoring (MRM) in the TIF samples (Table 2). Among these proteins, leucine-rich alpha-2-glycoprotein 1 (LRG1), tubulin beta-5 chain (TUBB5) and immunoglobulin J chain (IGJ) were confirmed to be stage-dependently increased in the serum of the mice [50]. Moreover, LRG1 and TUBB5 were also verified as potential biomarkers in the sera of patients with different CRC stages [50].

Similarly, Xie and colleagues used a widely accepted Apc−/+ mouse model of CRC whose pathological phenotype largely overlaps with human familial adenomatous polyposis and sporadic CRC [103]. The authors compared the TIF secretomes from Apc−/+ and WT mice of the different ages and identified 46 proteins with tumor progression-dependent expression profiles [51]. As reported in Table 2, the authors particularly focused their attention on six serine proteases and validated their levels by MRM assay, both in TIFs and in mouse sera. Importantly, levels of chymotrypsin-like elastase 1 (CELA1) and chymotrypsin-like protease (CTRL) were also validated by IHC in human TMAs containing 80 pairs of CRC tissues and their normal counterparts. Most importantly, the levels of CELA1, CTRL, chymotrypsin-like elastase 2A (CEL2A) and trypsin 2 (TRY2) were measured by MRM in the sera of CRC patients versus healthy controls and these proteins showed significantly higher expression in CRC sera. In particular, the best diagnostic performance was showed by the combination of CELA1 and CTRL which demonstrated a very high sensitivity and specificity [51].

## 5. Conclusions

There have been many advances in recent years in the search for tumor biomarkers using proteomic approaches, thereby allowing one to deeply analyze secretomes in several types of biological samples. In this context, proteomics emerged as a promising platform for the biological interpretation of secretome signatures underlying carcinogenesis, cancer progression and metastatic processes and for the identification of new cancer-related biomarkers with prognostic, diagnostic and predictive values.

The rationale of this review was to critically summarize the state-of-art of recent knowledge about the CRC secretome for biomarker discovery in terms of innovative technological and diagnostic performances. In fact, we provided a comprehensive overview of recent studies searching for CRC cell lines and tissue secretomes; particular attention was given to methodological aspects, technical limitations and relative precautions to avoid any bias, and to functional aspects of biomarker selection for validation in CRC patients. Moreover, we focused only on studies that selected and independently validated protein markers for early detection of CRC. 

This review highlights the great potential of cell line secretomes as valuable resources for identifying novel cancer-related secretory proteins. This aspect takes advantage of the possibility to use immortalized or primary cell lines as models representatives of the different grades of CRC differentiation and progression.

In technological terms, our review showed that secretome signatures of given CRC samples comprise several proteins, mainly secreted species, whose expression profiles, determined by label-free or labeling methods, need to be independently validated for further detection in CRC patients. In this scenario, we reported the findings of the recent secretome studies in order to help clinicians in candidate biomarker selection. ELISA and IHC are frequently the methods of choice for biomarker validations in serum/plasma and tissue samples, respectively. In addition to a limited number of CRC patients, the validations by antibody-based methods have some limitations related to the specificity of antibodies utilized and to the semi-quantitative analysis. Alternatively, DIA-MS approaches, such as SRM, MRM and SWATH-MS, are emerging as suitable methods for selection and verification of very promising diagnostic biomarkers, thereby allowing absolute quantitation and also increased sample size as required by rigorous clinical screening. 

As for biomarker discovery for CRC early diagnosis by secretome proteomics, this comprehensive overview points out the lack of common potential circulating biomarkers among the studies listed in Table 1 and Table 2. In addition to the heterogeneity of both sample types and experimental proteomic protocols, such a result could be explained by the different criteria adopted within each secretome study for the candidate selection. Hence, we cannot rule out the existence of a common set of putative circulating biomarkers if the complete datasets were compared. At this regard, bioinformatic analysis represents a mandatory step able to provide biological insights and to allow an integration/overlap with already published datasets, thereby favoring the selection of potential circulating biomarkers. To this aim, secretome databases should be freely-available and regularly updated based on the published untargeted proteomic studies. Despite these current limits, few candidates were successfully validated in CRC patients’ plasma, thereby raising hopes toward a complete translation in clinical practice.

## Figures and Tables

**Table 1:** Secretome studies done by using label-free proteomic approaches: identification of biomarkers for early diagnosis in colorectal cancer (CRC).

First Author [ref]	Year	Sample Types and Replicates				Pre-Mass Spectrometry (MS) Analysis ^1^	MS Platform	Quantitative Label-Free Analysis ^2^	Total Identified IDs	Potential Circulating Biomarkers
		Cell Line (CL)	CL Replicates	Tumor Tissue (TT)	TT Replicates					
Bernhard [31]	2013	LIM1215	*n* = 2 (each in triplicate)	TIF from LIM1215 xenografts in mice	*n* = 2	-	LC-MS/MS	-	39 LIM1215 5 LIM1215 TIF	CDH17 ^3^, LGALS3BP, PTK7
Imperlini [34]	2013	CaCo-2, HCT-GEO	*n* = 2 (technical)	-	-	10% SDS-PAGE	LC-MS/MS	SpC	176	CLU, ANXA5, PPIB, GPI, LGALS3BP, and SERPINE2 ^3^
Greening [35]	2013	LIM1215	n.r.	-	-	differential centrifugal ultrafiltration 4–12% SDS-PAGE or RP-HPLC	LC-MS/MS	SpC and TIC	987	COL12A1, COL4A2, LAMA3/5, LAMB1-3, LAMC1 ^3^/2, CSPG4, GPC1/4, HSPG2
Greening [36]	2013	LIM1215, LIM1863	*n* = 2	-	-	differential centrifugal ultrafiltration RP-HPLC	LC-MS/MS	SpC	474	FGFBP1, PLXDC2, DDR1, GPA33 ^3^, MACC1, SMAGP
Fanayan [45]	2013	panel of 21 CRC CL	n.r.	-	-	4–12% SDS-PAGE	LC-MS/MS	SpC	∼2500	SPTBN1, MSH2, MLH1, APC, NPM1, DEK, EZR, EGFR, MET, CDKN2A, SPTAN1, XPO4, LASP1, CEACAM5, CEACAM6 ^3^
Emmink [56]	2013	CSC	*n* = 3	-	-	NuPAGE Novex	LC-MS/MS	SpC	1254	ALDH1A1, BLMH
De Boeck [53]	2013	-	-	CAF	*n* = 2	4–20% SDS-PAGE	LC-MS/MS	-	412	TNC ^3^, LAMA2/4/5, MMP-2/3 ^3^, CTSH, GCP-2, CCL11, SDF-1, HGF ^3^, TIMP-4, SERPINA8, CALR
Shin [37]	2014	HCT-116, HCT-8	*n* = 3 (technical)	-	-	-	LC-MS/MS	-	898	TENA, PLOD3, FBLN4, SERPH, IPO5, PCBP2, NAP1L1, PTK7, RPSEP, TRFM ^3^, ASNS
Chen [54]	2014	-	-	CAF	*n* = 2 (each in duplicate)	10% SDS-PAGE	LC-MS/MS	-	230	-
de Wit [49]	2014	HT-29, CaCo-2, HCT-116, SW480, SW1398	-	CRC tissue	*n* = 4	4–12% SDS-PAGE	LC-MS/MS	-	2361 CL 2703 TT	AQR, COL12A1, DDX5, DNMT1, EXOSC8, FUBP1, HNRPDL, KHSRP, LCN2, MCM3, MCM5 ^3^, MCM6, NID1, RANGAP1, RRM1, SF3A3, SLK, SPTBN2, SSRP1, SUPT16H, TRIM28
Karagiannis [46]	2014	panel of 12 CRC CL	*n* = 3	-	-	-	LC-MS/MS	SpC	2979	GREM1, NME1, IGFBP7, CA9, LOXL2, VCAN, AZGP1, SRPX2, OLFM4 ^3^
Bukhari [44]	2015	HT-29	-	CRC patient plasma	*n* = 41 CRC patients *n* = 20 controls	IP 2DE	MALDI-TOF-MS/MS	-	3	VIM ^3^, KRT1, PPP1R16B
Lin [38]	2015	HCT-116, E1	*n* = 3 (technical)	-	-	MLAC enrichment	SWATH-MS	peak area extraction	568	GDF15, SPARC, SERPINE1, PLOD3, LAMB1 ^3^
Bozzi [55]	2017	-	-	Organoids	*n* = 2	-	LC-MS/MS	-	229	-
Basu [41]	2019	LS174T	n.r.	-	-	-	LC-MS/MS	n.r.	n.r.	CALCA, HADHB, MUC2, BGN, SMOC2, CTSD ^3^, VCAN, SEMA3B, ADPRHL2

^1^ The absence of a pre-MS analysis indicates a shotgun proteomic approach. ^2^ The absence of a quantitative method indicates a just qualitative label-free proteomic approach. ^3^ Validated by an independent experiment. n.r.: not reported in the paper.

**Table 2:** Secretome studies via labeling quantitative proteomic approaches: identification of biomarkers for early diagnosis in CRC.

First Author [ref]	Year	Sample Types and Replicates				Labeling Method	Pre-MS Analysis ^1^	MS Platform	Total Identified IDs	Potential Circulating Bionarkers
		Cell Line (CL)	CL Replicates	Tumor Tissue (TT)	TT Replicates					
Barderas [32]	2013	KM12SM KM12C	n.r.	-	-	SILAC	12.5% SDS-PAGE	LC-MS/MS	2087	PODXL ^2^, CD137L/TNFSF9 ^2^, VGF ^2^, SH3KBP1 ^2^, CTSS ^2^, LUM ^2^, NEO1 ^2^, SERPINI1 ^2^
Zeng [33]	2013	HT-29 NCM460	n.r.	-	-	SILAC	12% SDS-PAGE	LC-MS/MS	496 HT29 381 NCM460	VIM ^2^, IGFBP6 ^2^, GRN ^2^
Qiao [39]	2015	HT-29 SW620 LoVo	*n* = 3	CAF	*n* = 2	iTRAQ	-	2D LC-MS/MS	1114	COL6A3 ^2^
Wang [50]	2016	-	-	TIF from AOM-DSS mice	*n* = 4	iTRAQ	IPG-IEF	LC-MS/MS and LC-MRM-MS	776	COPA, HSP90AB1, GSS, VWA5A, SET, PRDX5, COTL1, S100A9, LRG1 ^2^, TUBB5 ^2^, IGJ ^2^
Xie [51]	2016	-	-	TIF from ApcMin/+ mice	*n* = 3	iTRAQ	-	LC-MS/MS and LC-MRM-MS	1174	CELA1 ^2^, CEL2A ^2^, CTRL ^2^, CTRB1 ^2^, TRY2 ^2^, TRY4 ^2^
Chen [40]	2018	HCT-116	*n* = 2	-	-	SILAC and dimethyl labeling	-	2D LC-MS/MS	772	LMAN2, PROS1, IGFBP6, LOXL2
Link-Lenczowski [42]	2019	CaCo-2	*n* = 3	-	-	anthranilic acid and 2-aminobenzamide labeling	HILIC- HPLC	MALDI-TOF-MS	77–82	H4N5F1 glycans

^1^ The absence of a pre-MS analysis indicates a shotgun proteomic approach. ^2^ Validated by an independent experiment. n.r.: not reported in the paper.
